# Presence of the blaTEM Gene in Commensal Neisseria spp.: A Possible Cause for the Acquired Drug Resistance Among Pathogenic Respiratory Bacteria

**DOI:** 10.7759/cureus.49389

**Published:** 2023-11-25

**Authors:** Anisha Sunil, Kennedy Kumar, Kopula S Sridharan

**Affiliations:** 1 Microbiology, Sri Ramachandra Institute of Higher Education and Research, Chennai, IND; 2 Laboratory Medicine, Sri Ramachandra Institute of Higher Education and Research, Chennai, IND

**Keywords:** pbp 2, β-lactamase, acquired drug resistance, commensal microbial flora, blatem gene

## Abstract

Background

The oral microbiome consists of various bacterial genera, with *Neisseria* spp. being a prominent part of this niche. While *Neisseria gonorrhoeae* and *Neisseria meningitidis* are human-restricted pathogens, non-pathogenic *Neisseria* species like *Neisseria sicca*, *Neisseria perflava, etc.,* are primarily commensals that can also behave as opportunistic pathogens. With increasing penicillin resistance in commensal *Neisseria*, there is a concern that these bacteria might harbor resistance genes that can be transferred to other pathogens. This study aimed to characterize the blaTEM gene (encodes for the plasmid-mediated β-lactamase enzyme that hydrolyzes the β-lactam ring) of commensal *Neisseria* spp. isolated from respiratory samples.

Methodology

The research was conducted in the Department of Clinical Microbiology at Sri Ramachandra University, Chennai. The specimens used were sputum and throat swabs, which were subjected to a series of phenotypic methods and matrix-assisted laser desorption ionization time-of-flight (MALDI-TOF) for speciation. The antibiogram was determined using the Kirby-Bauer disk diffusion method, and a PCR assay was utilized to identify the blaTEM_ _gene responsible for β-lactamase production.

Results

Out of 274 processed samples, 65 unique commensal *Neisseria* spp. were identified. The study highlighted the presence of the blaTEM* *gene in 93.9% (61) of the isolates, which is responsible for β-lactamase production. All isolates exhibited resistance to penicillin. Most blaTEM*-*positive commensal *Neisseria spp*. were susceptible to cefuroxime (83.6%), ceftriaxone (85.2%), and cefotaxime (85.2%). The high prevalence of the blaTEM gene in commensal *Neisseria* is alarming. The gene, found on plasmids, could potentially transfer to other related species like *Neisseria gonorrhoeae* and *Neisseria meningitidis*, as well as other Gram-negative bacilli.

Conclusion

The presence of resistance genes in commensal bacteria is of concern, as they might be reservoirs for resistance transfer to pathogenic strains. The study emphasizes the importance of continuous monitoring and deeper investigations into commensal bacteria, emphasizing the need for a broader community screening approach to understand resistance mechanisms in the normal microbiome.

## Introduction

The respiratory tract is divided into the upper and lower respiratory tracts. It is a well-known fact that upper respiratory is pooled with commensal microbial flora like Group A, C, G *Streptococci*, *Streptococcus pneumoniae*, other α-haemolytic *Streptococci*, non-typeable *Haemophilus influenza*, *Moraxella catarrhalis*, *Neisseria meningitidis*, commensal *Neisseria spp.*, *Staphylococcus aureus*, *Enterococcus* species, *Enterobacteriaceae*, some coliforms, and non-fermenters [1,2]. Potentially pathogenic organisms such as *Haemophilus*, *Mycoplasma*, and *Pneumococci* may also be found in the pharynx, along with Gram-negative anaerobes like *Prevotella*, *Porphyromonas*, *Fusobacterium*, and *Veillonella*. Gram-positive anaerobes like *Finegoldia*, *Parvimonas*, *Bifidobacterium*, *Eubacterium*, etc. Even though colonization of pathogens is prevented by a few natural protective mechanisms like the flow of saliva, secretary antibodies (IgA), lysozyme that participate in the destruction of bacterial cells, and the presence of normal flora (protective microorganisms), which produce substances that hinder successful invasion by harmful organisms and also act as physical barriers. The upper respiratory tract is often the site of initial colonization for the pathogens (*Neisseria meningitidis*, *Corynebacterium diphtheriae*, *Bordetella pertussis*, and many others) and is considered the first region of attack for these organisms. In contrast, the lower respiratory tract (small bronchi and alveoli) is usually sterile. If bacteria do reach these regions, they encounter host defense mechanisms, such as alveolar macrophages, that are not present in the pharynx [3,1].

Members of the genus *Neisseria* are gram-negative *diplococci*, which are usually the normal residents of the mucous membranes of mammals and reptilians; some species are primary pathogens for humans (*Neisseria gonorrhoeae* and *Neisseria meningitidis*). The commensal *Neisseria* are largely confined to the upper respiratory tract of humans. All organisms belonging to the *Neisseriaceae* are aerobic. They are oxidase and catalase-positive (except *Neisseria elongata*, which is catalase-negative). They colonize mucosal surfaces, generally without causing overt pathology, but may also cause infectious diseases when the host becomes vulnerable [4].

Drug resistance

Like other organisms, commensal *Neisseria* can also naturally transfer resistance genes to closely related species. They might constitute a DNA source for the emergence of antibiotic resistance in *meningococci*, as evident by the recent appearance of moderate resistance to penicillin in pathogenic *Neisseria*. The penicillin non-susceptibility is due to altered PBP2 (encoded by the penA gene) or, in other cases, to the production of a plasmid-encoded β-lactamase, causing high-level resistance [5]. Multi-resistant plasmids containing the TEM-1 β-lactamase have been isolated from oral commensal *Neisseria spp.* as well [6]. The spread of a potent ESBL from the commensal *Neisseria spp.* to the *Neisseria gonorrhoeae* population results in the degradation of ceftriaxone and the development of drug resistance in *Neisseria gonorrhoeae* [7].

In this pursuit, the current study was undertaken to look for the presence of the drug resistance gene among the commensal *Neisseria spp.* isolated from respiratory samples using phenotypic as well as molecular methods.

## Materials and methods

A cross-sectional study was conducted in the Department of Clinical Microbiology, Sri Ramachandra Laboratory Services (SRLS), Sri Ramachandra Institute of Higher Education and Research (SRIHER), Sri Ramachandra University, Chennai, after obtaining institutional ethics committee approval (CSP-MED/20/SEP/61/73). All the upper respiratory samples, consisting of sputum and throat swab clinical specimens, were subjected to Gram staining for the presence of Gram-negative *diplococci* and cultured on blood agar and chocolate agar plates. The plates were incubated overnight at 37°C. *Neisseria spp.* was phenotypically identified based on the growth and colony morphology on blood agar and chocolate agar plates. Biochemically, oxidase and catalase tests were used for the identification. Antibiotic susceptibility to various classes of antibiotics was determined using commercially procured antibiotic discs by the disc diffusion method in accordance with CLSI 2021 M-100 guidelines [8]. The antibiotics tested were penicillin (10 units), cefuroxime (30µg), ceftriaxone (15µg), tetracycline (30µg), cefotaxime (30µg), azithromycin (30µg) and ciprofloxacin (30µg). As per the CLSI 2021 M-100 guidelines for *Neisseria gonorrhoeae*, the results were interpreted as susceptible, intermediate, and resistant [8]. Polymerase chain reaction (PCR) for detection of the blaTEM gene was done using PureFast® Bacterial DNA minispin using primers obtained from HELINI Biomolecules. The primer used was TEM-F: TTTCGTGTCGCCCTTATTCC and TEM-R: ATCGTTGTCAGAAGTAAGTTGG at 260 bp.

## Results

**Figure 1:**
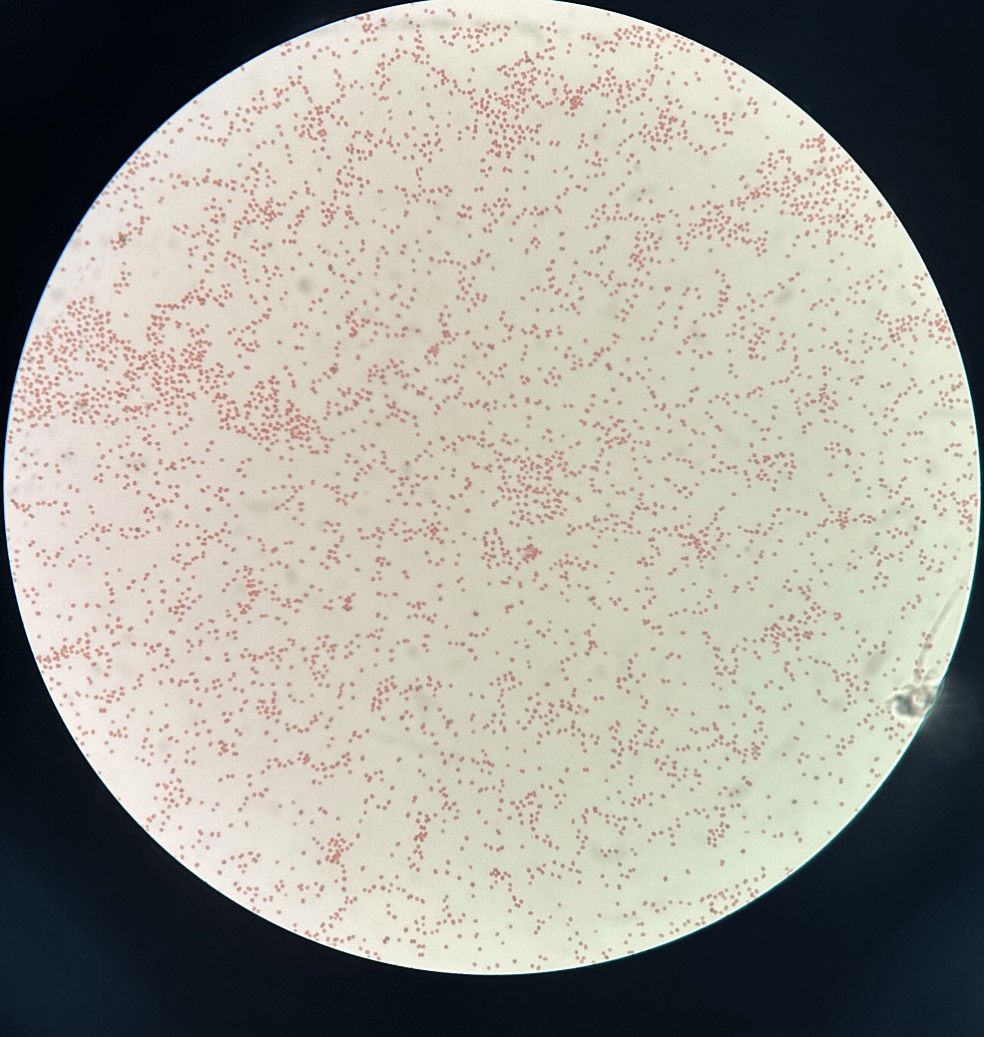
Neisseria spp. on gram stain (100x) appearing as gram-negative diplococci

**Figure 2:**
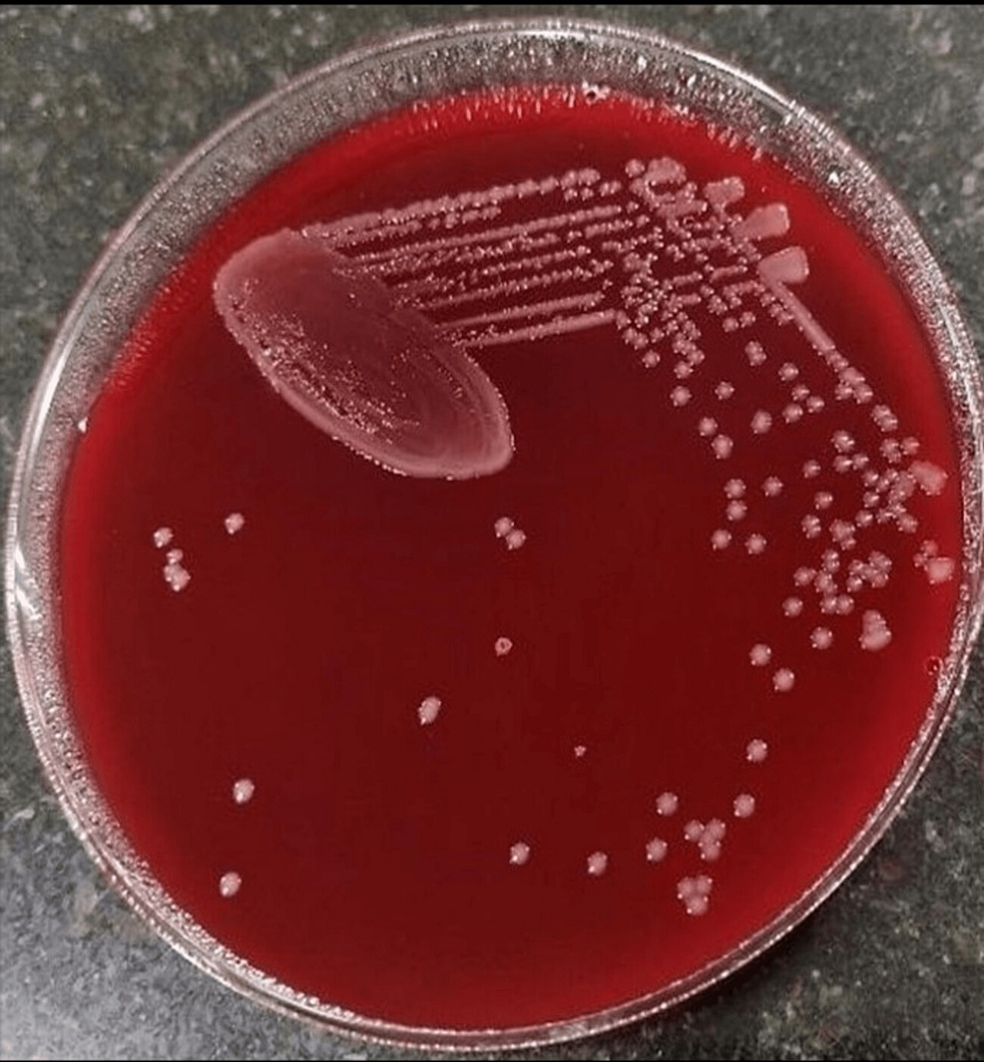
Growth of Nesseria on blood agar shows non-haemolytic colonies

**Figure 3:**
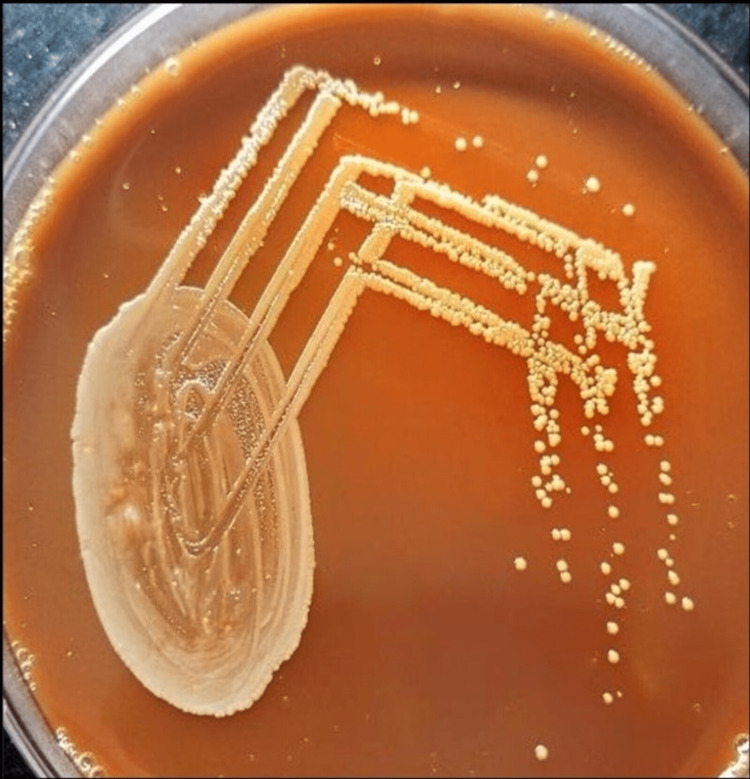
Growth of Nesseria on chocolate agar shows yellow-colored colonies

Of the 274 respiratory samples processed during the study period, 65 showed growth of commensal *Neisseria spp.* (Figures 1-3).

Out of the 274 samples that were processed during the study period, 238 (86.8%) were sputum samples, and the remaining 36 (13.2%) were throat swabs. Out of the 238 sputum samples, 54 (22.6%) of them had Neisseria spp., whereas in the throat swab, 11 (30.5%) out of 36 samples had Neisseria spp. Out of the 65 isolates, 40 (61.5%) were from males and 25 (38.5%) were from females. The highest number of Neisseria spp. isolates was in the age group of 61-70 years (23.1%), followed by in 51-60 years and 21-30 years (18.5%). The age-wise distribution of Neisseria spp. isolates is shown in Table 1.

**Table 1:** Age-wise distribution of the Neisseria spp. isolated in the study group

Age in Years	Total no of Neisseria spp. isolated
0-10	2
11-20	6
21-30	12
31-40	6
41-50	10
51-60	12
61-70	15
71 and above	2
Total	65

Among the 65 isolates, 31 (47.7%; n=65) were *Neisseria flava*/*subflava*/*perflava*, followed by 23 (35.4%) *Neisseria flavescens*, seven (10.8%) *Neisseria sicca*, three (4.6%) *Neisseria mucosa*, and one (1.5%) was identified as *Neisseria elongata*, which was catalase negative and appeared in coccobacillary form in the gram smear. Antibiotic susceptibility testing revealed that except for the four isolates that showed intermediate susceptibility to penicillin, all others were resistant to penicillin. *N. elongata* was found to be susceptible to all tested antibiotics except penicillin. Azithromycin had a higher susceptibility rate of 92.3%, followed by ceftriaxone and cefotaxime at 86.1% each. The details of the antibiotic susceptibility report for the *Neisseria* test isolates are shown in Table 2.

**Table 2:** Antibiotic susceptibility results of commensal Neisseria spp.

Antimicrobial agent	Disc Strength	Susceptibility percentage (No of isolates-65)
Penicillin	10 units	0% (0)
Cefuroxime	(30µg)	84.6% (55)
Ceftriaxone	(30µg)	86.1% (56)
Cefotaxime	(30µg)	86.1% (56)
Azithromycin	(15µg)	92.3% (60)
Tetracycline	(30µg)	80% (52)
Ciprofloxacin	(5µg)	81.5% (53)

**Figure 4:**
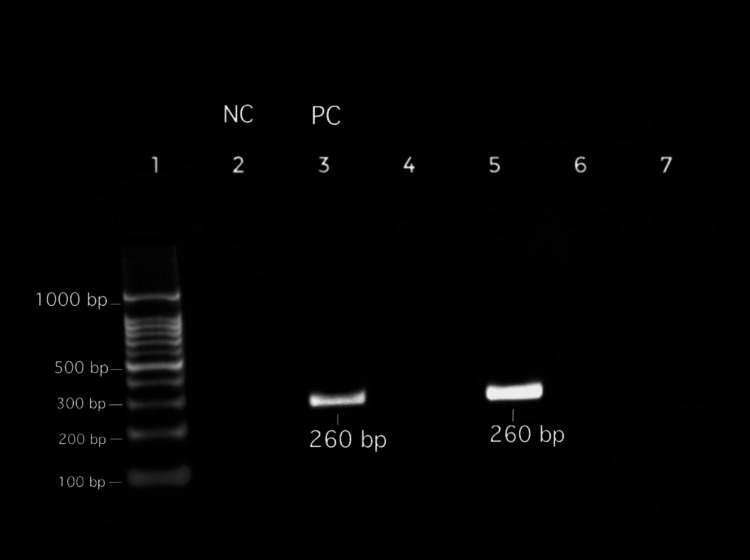
Gel electrophoresis of PCR for detecting the blaTEM gene (the band at 260 bp represents the presence of the blaTEM gene) Lane 1: 1000-bp ladder, Lane 2: Negative control, Lane 3: Positive control, Lane 4: Negative for blaTEM gene, Lane 5: Positive for the blaTEM gene, Lane 6: Negative for the blaTEM gene, Lane 7: Negative for the blaTEM gene

Polymerase chain reaction (PCR): PCR screening for blaTEM genes revealed 61 (93.9%; n=65) isolates had blaTEM genes in our study (Figure 4).

The remaining four (6.1%) did not harbor the blaTEM gene. The antibiotic susceptibility pattern in comparison with the presence or absence of the blaTEM gene is shown in Table 3.

**Table 3:** Comparison of antibiotic susceptibility among the blaTEM gene-positive and negative commensal Neisseria isolates

Antibiotics	bla_TEM _+ve (n=61)			bla_TEM _ -ve (n=4)	
	S	I	R	S	I
Penicillin	0 (0%)	-	61 (100%)	0 (0%)	4 (100%)
Cefuroxime	51 (83.6%)	-	10 (16.4%)	4 (100%)	-
Ceftriaxone	52 (85.2%)	-	9 (14.8%)	4 (100%)	-
Cefotaxime	52 (85.2%)	-	9 (14.8%)	4 (100%)	-
Azithromycin	56 (91.8%)	-	5 (8.2%)	4 (100%)	-
Tetracycline	48 (78.6%)	-	13 (21.4%)	4 (100%)	-
Ciprofloxacin	49 (80.4%)	5 (8.2%)	7 (11.4%)	4 (100%)	-

## Discussion

Of the total number of 274 samples, 65 (23.7%) grew commensal *Neisseria spp.* The colonization of *Neisseria spp.* was less in both age extremes (3.1% in both the less than 10 years and more than 71 years age groups). In our study, colonization by commensal *Neisseria* was observed after five years of age. The lesser number of colonizations in the older age group may be indicative of a poor oral milieu for the survival of commensal *Neisseria*, which needs further evaluation with a larger sample size. Early phenotypic studies done by Obi M.C. et al. in 1990 at Lagos, Nigeria, showed that the carriage of commensal *Neisseria spp.* in sputum samples was 28%, which was in concordance with our study, which had 22.6% of *Neisseria spp.* from sputum samples and 30.5% from throat swabs [9]. Based on our study, the throat swab seems to be a better specimen of choice for the isolation of commensal *Neisseria*. Mechergui et al. in 2014 [10] reported 100% correct identification of commensal *Neisseria spp.* by MALDI-TOF. In our study, we speciated commensal *Neisseria* both by conventional phenotypic methods and by mass spectroscopy and were able to identify five different species, namely *N. flava*/*subflava*/*perflava* complex, *N. flavescens*, *N. mucosa*, *N. sicca*, and *N. elongata*, both of which were comparable.

Various authors have reported different quantums of beta-lactamase production due to the presence of the blaTEM gene in commensal *Neisseria*, viz., Obi M.C. et al. [9] in 1990 at Lagos, Nigeria, showed 33% by the phenotypic method, and Mechergui et al. [5] of Tunisia in 2011 reported 9% by the genotypic method. In our study, 93.9% of isolates showed the presence of the blaTEM gene by PCR, a representative of beta-lactamase production, indicating that the increased usage of beta-lactam antibiotics of late may be the reason. However, about 86% of our isolates were susceptible to second and third generation cephalosporins, indicating that the blaTEM gene present in the isolates might not have been expressed and may be expressed in the presence of a substrate, i.e., cephalosporins. We have not estimated the detection of penA genes, which are responsible for penicillin resistance. 

A three-year study done in 2003 by Mechergui et al. [5] in Tunisia showed penicillin resistance of 34%, but our study shows penicillin resistance of almost 100%. Even a study done in Japan by R. Furuya et al. [11] between 2005 and 2006 showed 8.8% of penicillin resistance and 88.9% of intermediate resistance to penicillin by *N. subflava*. However, our *N. subflava* isolates showed 90.3% penicillin resistance, and 9.7% showed intermediate resistance. A Spanish study done by L. Arreaza et al. [12] in 2002 found that commensal *Neisseria* had a 100% intermediate susceptibility to penicillin for *Neisseria lactamica*. In 2015, Mechergui et al. [5] did antimicrobial susceptibility testing for commensal *Neisseria spp.* in Tunisia, which reported 87% resistance to penicillin and the rest of the isolates to be intermediately susceptible to penicillin, which correlates with our findings.

One of the studies done in New York, USA, by Michael A. Fiore et al. [13] in 2020 showed penicillin resistance of 19.2%, followed by cefixime resistance of 11.9% and ceftriaxone resistance of 7.6%. In our study, we found 13.9% resistance to 3rd-generation cephalosporins and almost 100% resistance to penicillin. The same study showed ciprofloxacin resistance of 7% and tetracycline resistance of 14%, respectively. In our study, we had ciprofloxacin resistance of 18.5% and tetracycline resistance of 20%, respectively, which contrasts with the Japanese study done by R Furuya et al. [11], which showed 44.4% intermediate resistance and 31.1% resistance to ciprofloxacin and 60% intermediately resistant and 28.9% resistant to tetracycline in *N. subflava* isolates. In our study, the *N. subflava* isolates showed 16.13% resistance and 6.45% intermediate resistance to ciprofloxacin. A study done by Michael A. Fiore et al. [13] in 2020 in New York, USA, showed ciprofloxacin resistance to be 15.4%, which is in concordance with our study. The same study had macrolide resistance of about 42.3%, but our study showed 35% resistance to erythromycin; however, we have also tested for azithromycin susceptibility as per CLSI 2021, which showed a resistance of 7.7%. Table 4 shows the comparison of the commensal *Neisseria spp.* antibiotic susceptibility among the various studies conducted.

**Table 4:** Comparison of commensal Neisseria spp. antibiotic susceptibility with various studies conducted

Study	Place & Year of the Study	Antimicrobial Microbial Resistance %				
		Penicillin	3^rd^ Generation Cephalosporin	Macrolides	Tetracycline	Ciprofloxacin
Our Study	India 2020-2021	94%	13.9%	7.7% (azithromycin)	20%	18.5%
Mechergui et al.	Tunisia 2003	34%	-	-	-	-
Mechergui et al.	Tunisia 2014	87%	-	35% (erythromycin)	-	-
R Furuya et al.	Japan 2005-06	8.8%	-	-	28.9%	31.1%
Fiore et al.	USA 2020	19.2%	7.6%	42.3% (azithromycin)	-	-
L Arreaza et al.	Spain 2002	0%	-	-	14%	7%

Drug resistance gene

Our study had 93.9% blaTEM by conventional PCR. However, the Tunisian study showed the presence of blaTEM in 9% of their isolates [5]. In this study, the identification of the TEM-1 gene in 93.9% of commensal *Neisseria* is alarming, as it is well proven that the resistance gene can horizontally transfer to pathogenic *Neisseria* (*Neisseria gonnorhoea* and *Neisseria meningitidis*), thus making the previously susceptible pathogenic strains into resistant strains. The possible reason for acquiring resistance could be because of the irrational and indiscriminate use of antibiotics in poultry feed [14].

The limitations of the study are screening of 274 samples had only isolation of 65 commensal *Neisseria spp.* Inclusion of a greater number of commensal *Neisseria spp.* would have been better for more information, and also the clonal relatedness of blaTEM gene between the commensal *Neisseria spp.* and the pathogens is not performed.

## Conclusions

The possibility of the blaTEM gene, which is present in the plasmid of commensal *Neisseria*, getting transferred to other closely related species like *Neisseria gonnorhoea* and *Nesseria meningitidis*. This could be one of the prime reasons for acquiring drug resistance among the pathogenic bacteria, especially in the hospital environment. A few SNPs (single nucleotide polymorphisms) in the beta-lactamase TEM gene will convert them into extended-spectrum beta-lactamases and give them resistance to other classes of antibiotics as well. The source of blaTEM in commensal *Neisseria* is yet unknown. A community screening for the presence of the blaTEM gene may shed light on this state. According to our study, a throat swab seems to be a good specimen for screening blaTEM in commensal bacteria. Since most of the studies are being done on pathogenic *Neisseria*, a large multi-center study in commensal *Neisseria* may give us hints for explaining the source of resistance mechanisms present in normal microbiomes transferring to probable pathogenic organisms.
